# Targeting triple negative breast cancer stem cells using nanocarriers

**DOI:** 10.1186/s11671-024-03985-y

**Published:** 2024-03-07

**Authors:** Nagasen Dasari, Girija Sankar Guntuku, Sai Kiran S. S. Pindiprolu

**Affiliations:** 1 Andhra University College of Pharmaceutical Sciences, Andhra University, Vishakhapatnam, Andhra Pradesh India; 2 Aditya Pharmacy College, Surampalem, Andhra Pradesh India; 3 Jawaharlal Nehru Technological University, Kakinada, Andhra Pradesh India

**Keywords:** Breast cancer stem cells, Molecular targets, Nano carriers, Anti-BSCS agents

## Abstract

Breast cancer is a complex and heterogeneous disease, encompassing various subtypes characterized by distinct molecular features, clinical behaviors, and treatment responses. Categorization of subtypes is based on the presence or absence of estrogen receptor (ER), progesterone receptor (PR), and human epidermal growth factor receptor 2 (HER2), leading to subtypes such as luminal A, luminal B, HER2-positive, and triple-negative breast cancer (TNBC). TNBC, comprising around 20% of all breast cancers, lacks expression of ER, PR, and HER2 receptors, rendering it unresponsive to targeted therapies and presenting significant challenges in treatment. TNBC is associated with aggressive behavior, high rates of recurrence, and resistance to chemotherapy. Tumor initiation, progression, and treatment resistance in TNBC are attributed to breast cancer stem cells (BCSCs), which possess self-renewal, differentiation, and tumorigenic potential. Surface markers, self-renewal pathways (Notch, Wnt, Hedgehog signaling), apoptotic protein (Bcl-2), angiogenesis inhibition (VEGF inhibitors), and immune modulation (cytokines, immune checkpoint inhibitors) are among the key targets discussed in this review. However, targeting the BCSC subpopulation in TNBC presents challenges, including off-target effects, low solubility, and bioavailability of anti-BCSC agents. Nanoparticle-based therapies offer a promising approach to target various molecular pathways and cellular processes implicated in survival of BSCS in TNBC. In this review, we explore various nanocarrier-based approaches for targeting BCSCs in TNBC, aiming to overcome these challenges and improve treatment outcomes for TNBC patients. These nanoparticle-based therapeutic strategies hold promise for addressing the therapeutic gap in TNBC treatment by delivering targeted therapies to BCSCs while minimizing systemic toxicity and enhancing treatment efficacy.

**Graphical abstract:**

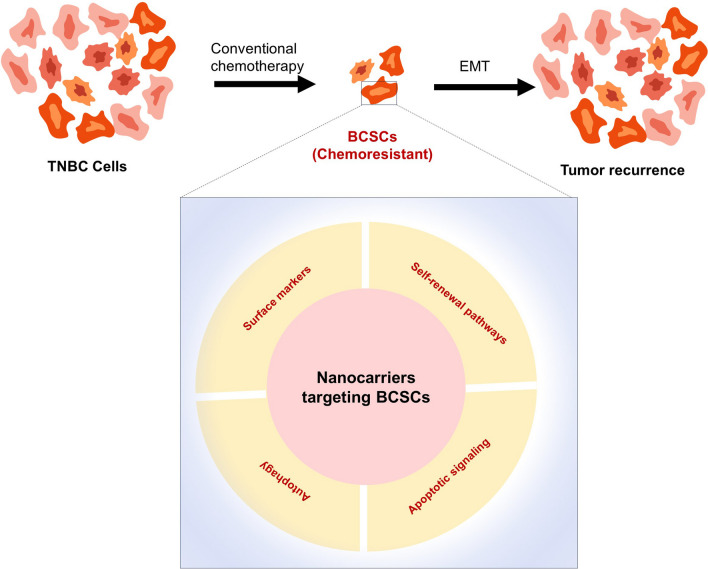

## Introduction

Breast cancer is a common and highly prevalent cancer subtype globally according to world health organization [1–3]. Breast cancer has been identified as a significant contribution to mortality rates trailing closely behind lung cancer. Based on the most recent data available around 2.5 million women were diagnosed with breast cancer annually resulting in approximately 685,000 deaths world-wide [4].

TNBC constitute around 20% among all breast cancer cases and it is characterized by the absence of ER, PR, and HER2 receptors. It presents a highly aggressive phenotype, tends to metastasize more, and frequently acquires resistance to chemotherapy. As per reports in 2023, a substantial number of cases of TNBC were recorded, reaching approximately 2,088,849. These statistics highlight the significant prevalence of TNBC as a type of cancer affecting women. According to available treatment data, TNBC has a mean survival rate of approximately 10.2 months. For cases where the tumour is localized in regional areas, the 5-year survival rate reaches 65%. However, when the tumour has metastasized to distant organs, the survival rate drops significantly to 11% [1, 5–7]*.* Metastasis, chemoresistance, relapse are the major challenges in TNBC treatment [8–13]. The presence of cancer stem cells (CSCs), which contribute to tumor initiation, progression, metastasis, and recurrence. These cells possess self-renewal capabilities, resist conventional therapies, and can regenerate the heterogeneous cell populations within tumors. Several challenges are associated with treating BSCS include Heterogeneity, Resistance to Therapy, Microenvironment Interactions, Identification and Isolation and Therapeutic Resistance Mechanisms.

**Fig. 1:**
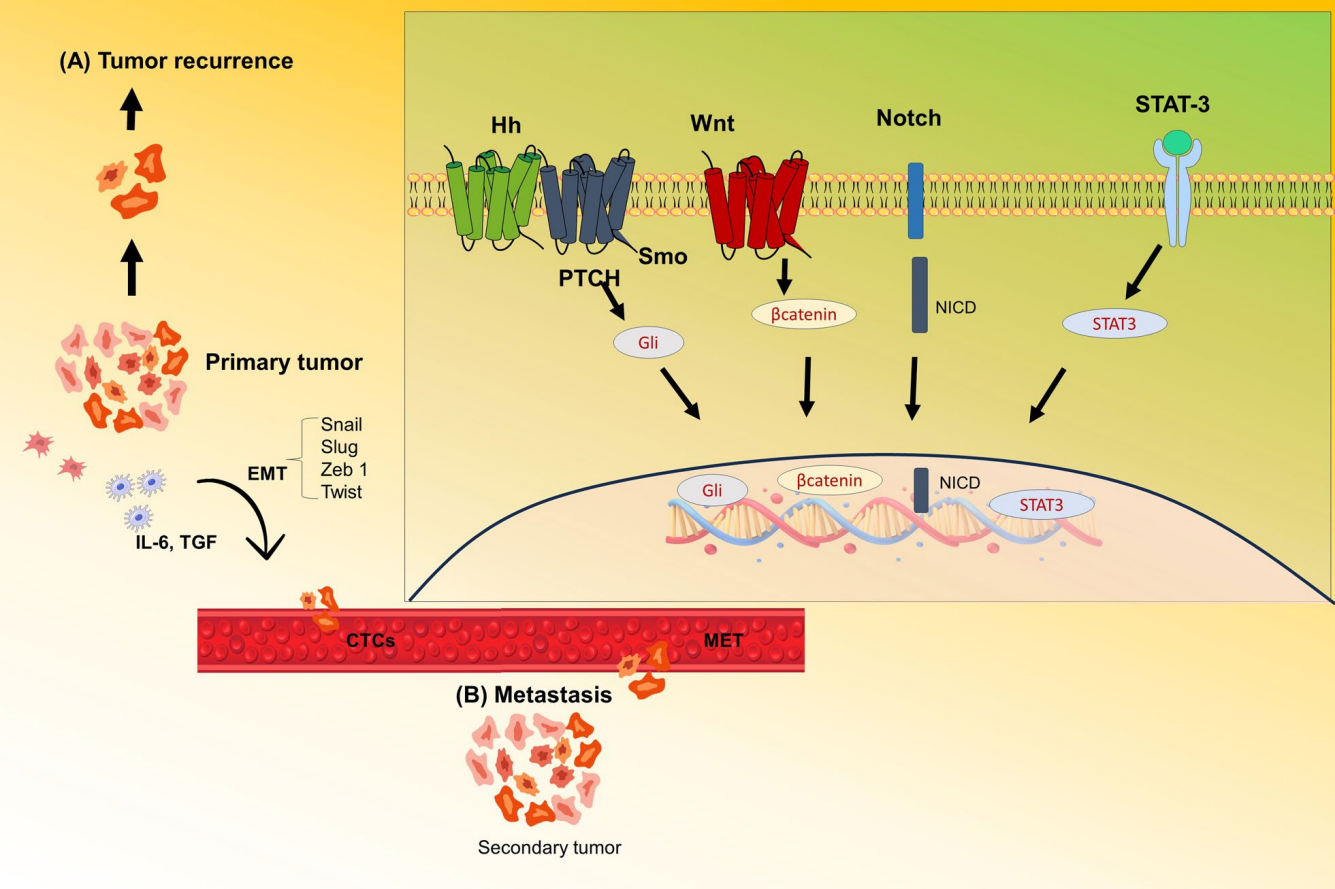
The role of cancer stem cells in Tumour recurrence & Transmission. **A** Conventional therapy for elimination of non-tumour stem cells & stem cells and results in tumour relapse; **B** The activation of transcription factors, for epithelial-to-mesenchymal transition (EMT), in BCSCs or metastatic cells. This phenotypic change facilitates their dissemination and migration. Cells undergoing EMT acquire the ability to enter the bloodstream, evading immune surveillance, and eventually reach target organs. Once in the specific organ site, these cells can enter a Inactive state that can last for months to decades. Alternatively, they can endure mesenchymal-to-epithelial transition (MET), reverting back to an epithelial state, and initiate the growth of secondary tumors. **C** Essential Signaling Pathways Associated with the Survival of BCSCs [14]

The Prescence of Breast cancer stem cells (BSCS) within TNBC have garnered notable focus owing to their distinctive possibility for self-renewal and their involvement in the formation of tumours in distant organs and tissues. These BCSCs display quiescence, exhibit elevated levels of drug efflux carrier proteins and, and possess robust “DNA repair mechanisms”, rendering them less susceptible to programmed cellular death triggered by chemotherapy drugs. The survival of a pool of BCSCs following conventional chemotherapy can lead to the emergence of future tumours and subsequent relapse. Additionally, BCSCs have a crucial function in promoting metastasis by facilitating epithelial-to-mesenchymal transmission (EMT). This process entails the disturbance of cellular cohesion in epithelial cells and the attainment of invasive and migratory characteristics, crucial for the spread of cancer to distant locations (Fig. 1).

Eliminating BCSC’s is therefore necessary to provide radical cure of TNBC. Surface markers, such as CD44 and CD133 key signalling pathways like Notch, Wnt, and Hedgehog, are the key targets for elimination of BSCS. Another approach involves promoting the differentiation of BCSCs into non-BSCS, thereby enhancing susceptibility to conventional cancer treatments. Additionally, exploiting the unique metabolic characteristics of BCSCs, such as their reliance on pathways like glycolysis, offers a promising strategy for inducing cell death. Immunotherapy plays a crucial role by activating the immune system through checkpoint inhibitors or CAR T-cell therapy to recognize and eliminate BCSCs. Further, Epigenetic modulation aims at altering the regulation of BCSCs, controlling their self-renewal capacity, and enhancing sensitivity to standard treatments are the approaches that are being widely researched for targeting BSC’s.

In recent years various therapeutic agents were proposed for targeting BSCS. However, the therapeutic effectiveness of these anti-BCSC compounds is limited due to low aqueous solubility, brief circulation duration, variable stability and non-specific side effects. Addressing these issues necessitates the evolution of suitable drug delivery system aimed at augmenting bioavailability and specifically target BCSC without causing non-specific effects. Recently nano based carriers have emerged as encouraging carriers for targeting BCSCs, offering site-specific delivery, improved drug bioavailability, and enhanced stability [15–17]. In the present review we discuss the applications of various nanocarriers for targeting BSCS in TNBC [18, 19].

Nanotechnology based approaches are being employed for the diagnosis and treatment of TNBC. Nanosystems have a significant role in the study of the interaction of malignant cells with their microenvironment through receptor-based targeted approach. Nowadays, lipid-based nanocarriers are being popularized in the domain of pharmaceutical and medical biology for cancer therapy. Lipidic nanoparticlized systems (LNPs) have proven to have high loading efficiency, less toxicity, improved therapeutic efficacy, enhanced bioavailability and stability of the bioactive compounds compared to traditional drug delivery systems. Several LNPs based formulations have been undertaken in various phases of clinical trials in different countries. This review highlights the importance of chemotherapeutics based lipidic nanocarriers and their anticipated use for the treatment of BC. Furthermore, the clinical trials and future prospective of LNPs have been widely elaborated [20–23].

With the advances in nanomedicine and drug delivery, this review briefly focused on various modes of nanodrug delivery including Lipid based Nanoparticles (nano emulsion, Liposomes, Lipoidic micelle, Solid lipid nanoparticles, Nanostructured lipid carrier), Polymer based nanoparticles (Polymeric nanoparticles, polymeric micelle, polymer drug conjugate, dendrimer), and Inorganic nanoparticles (Iron oxide nanoparticles, gold nanoparticles, carbon nanotubes, quantum dots). Nanocarrier targeting is advantageous due to minimum systemic toxicity. Several nanomedicines have been approved and marketed so far, liposomal preparation of daunorubicin as DaunoXomeVR and vincristine as Onco-TCSVR and many more under clinical trials.

The FDA approved PEGylated liposomal formulation under the name of (DoxilVR) contains anti-cancer agent doxorubicin. Novel nanomedicines offers compromising drug design and fabricating formulations in fast track based on cancer homo or heterogenic profile and make treatment mode quite effective and rationale. The successful delivery of nanocarrier entirely depends on the physicochemical characterization such as particle size, surface charge, density, surface topography and physiological condition of target site. The engineered functionalized nanoparticles (NPs) depicting various ligands for conjugation to NP surface through linker. Active and passive are broadly two common modes of drug targeting using nanocarriers. Through passive mode of drug transport nanocarriers deprived of cell recognition and accumulates near tumour microenvironment give the so-called enhanced permeation and retention effect (EPR). Active targeting on the contrary is site-specific delivery of nanocarrier that is capable of cell recognition by binding with membrane receptor of cells or tissues. The surface activation or modifications of nanocarrier accomplished by monoclonal antibodies or ligand are necessary steps taken before active targeting. Such functionalized nanocarrier has high affinity to get internalized in tumour cells via distinguished cellular pathways. In the active mode of targeting approach, two types of cell target are possible: (a) tumour cell targeting mediated via cell surface receptor and (b) tumour endothelial cell targeting is common.

Targeting tumour cell mediated via cell surface receptor: In this mode of nanocarrier targeting, the primary aim is successful delivery of nanocarrier into cancer cell through receptor opted (over-expressed receptor on cell surface) ligand- gated internalization thus improving enhanced uptake of nanocarrier e.g. higher uptake of macromolecular drugs protein, DNA, and siRNA, etc.

Tumour endothelial cell targeting: The endothelial drug targeting has been much appreciated and effort has been made for the development of anti-angiogenic agent. The growth of tumour largely depends on the blood supply to the growing cells. The disruptions of tumour vasculature remain a goal for scientist that apparently focused and targeting such areas are exciting therapeutic opportunities. The targeting endothelial cells are an effective way of delivering nanomedicine for anti-tumour efficacy.

There are many challenges for delivering the drug to tumour microenvironment site in breast cancer particularly TNBC. The challenges are tumour heterogenicity, Extracellular Matrix (ECM) Barriers, Hypoxia and Acidic pH, Drug Efflux Pumps and Resistance Mechanisms, Immune Suppression [22]. Nanocarriers offer a promising approach to overcoming drug delivery challenges in the tumor microenvironment of breast cancer, including triple-negative breast cancer (TNBC). Engineered with specific physicochemical properties and surface modifications, nanocarriers can facilitate enhanced penetration into dense tumor tissue while minimizing off-target effects.

**Fig. 2:**
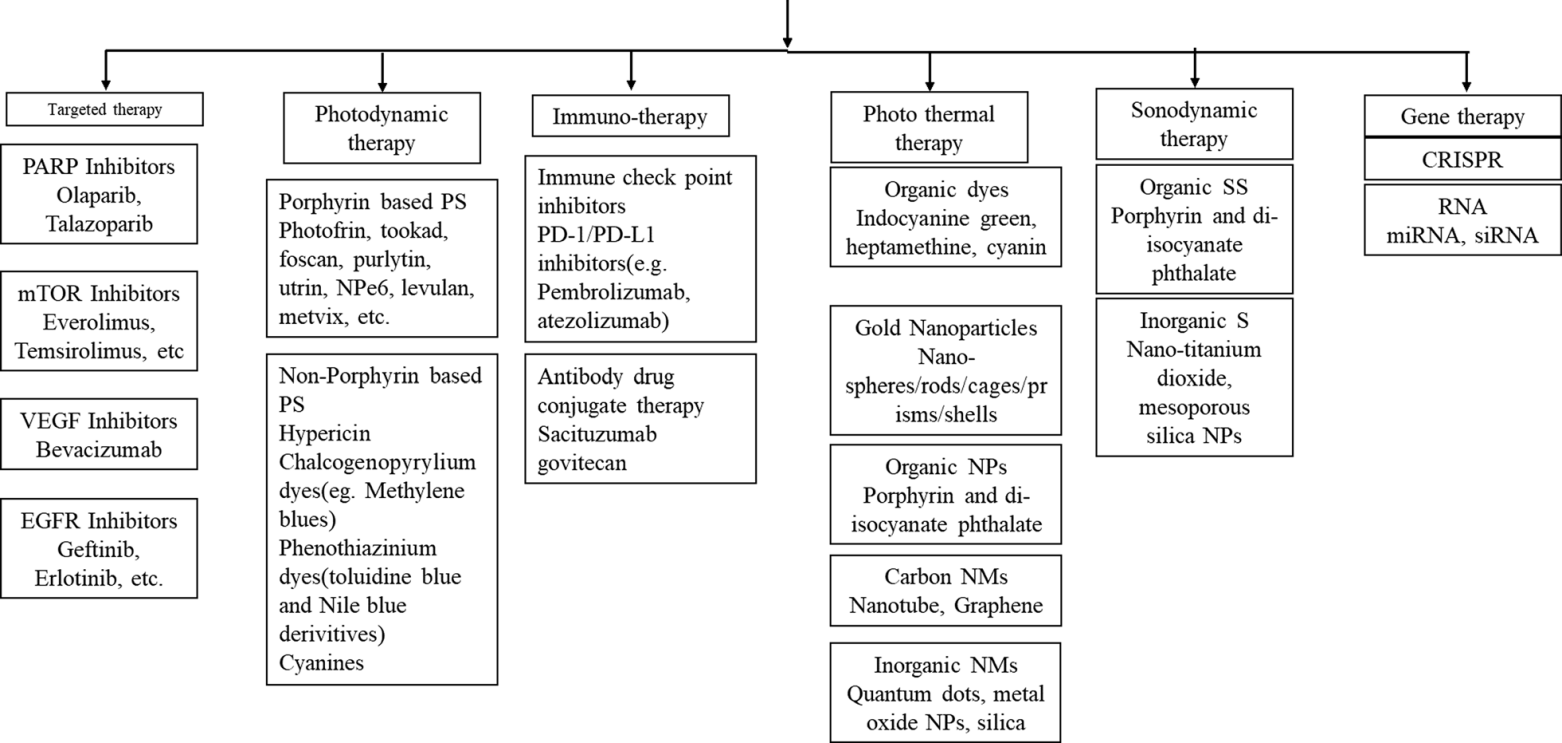
Therapies Against TNBC

Therapeutic strategies for TNBC treatment: Various Therapeutic approaches utilized for TNBC, include conventional methods like chemotherapy, surgery, radiation. However, chemo/radio resistance BCSC mediated relapse are the major limitations in Fig. 2.

Further, conventional chemotherapy can cause side effects due to its impact on both cancerous and normal cells. High doses of chemotherapeutic agents may result in anaemia, discomfort, nausea, vomiting, hair loss, weight fluctuations, fatigue, and nervousness. The treatment of TNBC, in particular, lacks specific targets due to its heterogeneous nature and a range of pathological features. Nevertheless, there are several chemotherapy treatment protocols used, including neoadjuvant therapy, to address TNBC [8, 13, 37].

In recent years, researchers proposed various therapeutic modality to overcome limitations associated with conventional chemo/radio therapy. In this section we discuss these for effective targeting of TNBC modalities [38–41]. To effectively treat this, targeting molecular pathways at a molecular level. Molecular therapeutics offer the capability to modify the activity of upregulated receptors, Polypeptides, chemical messengers, or DNA repair mechanisms. Various targeted therapies have emerged as promising options for TNBC treatment, providing increased selectivity and efficacy.

### Molecular targeted therapies

Targeted therapy operates at the molecular level, by targeting specific enzymes or cellular mechanisms implicated in cell division, autophagy, and apoptosis. These include PARP inhibitors (“poly (ADP-ribose) polymerases”), mTOR inhibitors (mammalian target of rapamycin), VEGF inhibitors (vascular endothelial growth factor receptor), and EGFR inhibitors (Epidermal growth factor receptor). For example, PARP inhibitors are drugs that target “poly (ADP-ribose) polymerases” (PARPs), enzymes accountable for ADP-ribose transfer to protein targets. PARPs play vital functions in different cellular activities, like “chromatin structure modulation”, “transcription”, “replication”, “recombination”, and repairing DNA. By inhibiting PARP using drugs like Olaparib and talazoparib, DNA replication is disrupted through PARP DNA entrapment, leading to replication stress-induced mitotic catastrophe and ultimately inducing cell death [11, 42, 43]. PARP plays a vital role in repairing single-strand breaks in DNA, while germline BRCA1/BRCA2 mutant genes function as Cancer-inhibiting proteins involved in repairing double-strand breaks. By inhibiting PARP, the DNA repair mechanism in tumour cells is disrupted, leading to DNA damage and the arrest of DNA replication. PARP inhibitors have shown promising clinical efficacy in TNBC patients, particularly when administered alongside immunotherapy. This combination has demonstrated improved outcomes, including prolonged existence and enhanced quality of existence.

The use of PARP inhibitors and EGFR-targeted nanoparticles represents potential remedy approaches for the treatment of TNBC, offering possible advantages concerning to DNA repair inhibition and targeted drug delivery. The use of PARP inhibitors and EGFR-targeted nanoparticles represents potential remedy promising therapeutic approaches for the treatment of TNBC, offering possible advantages concerning to potential benefits in terms of DNA repair inhibition and targeted drug delivery.

The monoclonal anti-VEGF-A antibody, bevacizumab, which Inhibits blood vessel formation, has not yet demonstrated consistent clinical evidence for its efficacy in TNBC. In approximately 70% of TNBC cases, the epidermal growth factor receptor is overexpressed, resulting in poor prognosis and resistance to conventional chemotherapy. Combination therapy with chemotherapeutics and EGFR inhibitors has shown a synergistic effect, providing better outcomes. EGFR is a critical protein participating in cell division and dissemination of TNBC. Targeted delivery of nifetepimine, an EGFR inhibitor, using “poly (lactic-co-glycolic acid)” nanoparticles has been investigated. These EGFR-targeted nanoparticles have demonstrated effective apoptosis induction in “MDA-MB-468” cells in vitro, as well as extended life expectancy rates, diminished tumour bulk, and enhanced bioavailability of nifetepimine in TNBC.

mTOR Plays a central role in controlling cell multiplication, autophagy, and apoptosis by influencing diverse signalling pathways. Therapeutic drugs, like Everolimus and temsirolimus, have been developed as mTOR inhibitors. These inhibitors have demonstrated the capacity to enhance the efficiency of hormone therapy and effectively hinder cell proliferation in TNBC cells. Through directing mTOR, these drugs disrupt aberrant signalling pathways and exert control over the growth of TNBC cells (discuss about Beta catenin, notch etc.).

Tumour angiogenesis is a critical process in cancer development and advancement. VEGF has been recognized as significant prognostic marker in breast cancer patients. Inhibiting this, as exemplified by the drug Bevacizumab, can effectively impede tumour cell development and progression. EGFR is a proto-oncogene involved in cell proliferation and survival. Investigation indicates that the Excessive expression of “ubiquitin-associated and SH3 domain-containing B” is linked to TNBC, promoting malignant growth, invasion, and metastasis by modulating EGFR. Hence, the use of EGFR inhibitors like Gefitinib and Erlotinib can aid in preventing metastasis and retard the growth of TNBC cells [44–46]. PARP inhibitors disrupt DNA repair mechanisms, while EGFR inhibitors specifically target the overexpressed EGFR in TNBC. VEGF inhibitors, on the other hand, act as anti-angiogenic agents, inhibiting the growth of blood vessels that supply nutrients to tumour cells. Combining VEGF inhibitors with chemotherapeutic agents has shown enhanced efficacy against TNBC. Ongoing clinical investigations are exploring other promising combinations of molecular agents for TNBC treatment. By targeting specific molecular pathways, these therapies aim to inhibit metastasis and improve outcomes for patients with TNBC (Table 1).

**Table 1:** Mechanism of action of various anti-BSC agents

Anti-BCSC agent	Mechanism of action	References
Salinomycin	By selectively targeting and eliminating cancer stem cells through disruption of various signalling pathways, including Wnt/β-catenin and STAT3	[24]
Simvastatin	By inhibiting the mevalonate pathway, disrupting lipid metabolism	[25]
Flubendazolum	By disrupting microtubule dynamics and inhibiting Wnt/β-catenin signalling	[26]
Everolimus	By inhibiting the mTOR pathway	[27]
Niclosamide	By disrupting Wnt/β-catenin signalling and inducing mitochondrial dysfunction	[28]
Metformin	Effects through AMPK activation, mTOR inhibition	[29]
Tranilast	By inhibiting TGF-β signalling and suppressing pathways	[30]
Disulfiram	By inhibiting ALDH activity, chelating copper, inducing ROS production, and modulating signalling pathways	[31]
Epigallocatechin gallate analogs	By interfering with signalling pathways and cellular processes critical for cancer stem cell survival and self-renewal	[32]
Thioridazine	Through modulation of the Hedgehog signalling pathway and inhibition of cancer stem cell self-renewal	[33]
Cyclopamine	By inhibiting the Hedgehog signalling pathway	[34]
Sacituzumab govitecan	A humanized monoclonal antibody hRS7 targeting trophoblast cell surface antigen 2, linked to the topoisomerase I inhibitor SN-38 by a hydrolysable linker	[35]
Polyphenols (resveratrol, quercetin, kaempferol, etc.)	Also exhibit synergistic effects with chemotherapy drugs, making them involve the modulation of several signalling pathways, such as PI3K/Akt, MAPK, STATT, and NF-κB pathways	[12]
Tetra hydro iso quinoline derivative	A potent and selective CDK9-cyclin T1 inhibitor, by targeting the ATP binding site, compound 1 not only inhibited CDK9 activity but also disrupted the CDK9-cyclin T1 protein–protein interaction	[36]

### Photo dynamic therapy (PDT)

The method which utilizes light energy to selectively destroy proliferating cancer cells through the production of reactive oxygen species (ROS) and Temperature. PDT is categorized into two main types: based on porphyrin and based on non-porphyrin-. Porphyrin-based PDT involves bonding porphyrins with anticancer active substances such as Photofrin, Tookad, Foscan, Purlytin, Metvix, and Levulan. These complexes enable targeted drug delivery to tumour cells, reducing toxic effects on normal tissues and achieving a combined effect of chemotherapy and PDT. On the other hand, non-porphyrin-based PDT includes examples like Hypercin, Phenothiazinium (e.g., Toluidine), Phenothiazinium dyes, and Chalcogenopyrylium dyes, where anticancer drugs are not bonded with porphyrins. Both porphyrin-based and non-porphyrin-based PDT offer promising strategies for selectively destroying cancer cells while minimizing side effects on normal tissues. Different researchers have developed nano formulations for improving the therapeutic efficacy of anti-cancer agents like carbon nanotubes, solid lipid nanoparticles, lipid drug conjugates, polymeric nanoparticles, lipidic micelles etc. nanoparticles reduces the off-target effects of encapsulated anticancer agents and improves their anticancer effects. The nanocarriers travel by using external light energy to reach the destination of targeted site to destroy the oncogenes [47–49]. In a research work carried out by sifa dogan et al., The cytotoxicity of CoPc-COOH in MDA-MB-231 cells was evaluated after activation of phthalocyanine derivative CoPc-COOH with RED light. Apoptotic effect of single CoPc-COOH, RED light, and their combinations were examined by using the Annexin V method and the ROS generation potential using the carboxy-H2-DCFH-DA test in flow cytometry [50]. In another study carried out by Yujing Wang et al., designed a luminescent monofunctional platinum(II) complex with BODIPY derivative in green light (520 nm). Besides, the anticancer activity it also help DNA repair and induce p53-mediated apoptosis of TNBC cells [51].

### Immunotherapy

Immunotherapy is categorized into two main types: inhibitors of immune checkpoint and drug conjugate therapy of antibody. Immune checkpoint inhibitors, such as Pembrolizumab and Atezolizumab, target molecules like PD-1/PD-L1 to activate T cells and empower them to effectively eliminate tumour cells. On the other hand, antibody–drug conjugate therapy utilizes monoclonal antibodies like Sacituzumab and Govitecan. These antibodies specifically recognize and destroy cancer cells by binding to their unique antigens. Both approaches, immune checkpoint inhibitors and antibody–drug conjugate therapy, are effective strategies in harnessing the immune system to combat cancer [52, 53]. In research conducted by Yi Zheng et al., reported report a combined tumor-therapeutic strategy based on Prussian blue (PB)-mediated photothermal therapy with Mn2 + -augmented immunotherapy by synergistically activating the cGAS-STING pathway. This work represents non-invasive Mn-based tumor-immunotherapeutic modality for immunotherapy of metastatic-prone tumors like TNBC [54].In another investigation led by Rakshmitha Marni et al., as the potential therapy response markers for the antiviral drug 2-thio-6-azauridine (TAU) in proteomic profiling and ROC analysis to identify CD151 as potential therapy response markers against TNBC [55]

### Photothermal therapy (PTT)

This method employs electromagnetic radiation, specifically in the infrared wavelengths, to target and treat cancer. It involves activating a photosensitizer with specific light wavelengths. Various substances can act as photosensitizers, such as organic dyes (e.g., Indocyanine Green and Heptamethine Cyanin), gold nanoparticles (nanospheres, rods, cages, prisms, and shells), organic nanoparticles (semiconducting polymeric nanoparticles and porphysomes), carbon nanomaterials (nanotubes and graphene), and inorganic nanomaterials (quantum dots, metal oxide nanomaterials, and silica nanoparticles). Through these substances, nanocarriers can be precisely guided to tumour cells using electromagnetic radiation. This targeted approach enables the selective destruction of tumour cells while minimizing harm to healthy tissues [56–58]. In a research work carried out by Asif Mohd Itoo et al., developed a 2D carbon nanomaterial graphene oxide (GO)-based nanoplatform that converted to 3D colloidal spherical structures. The therapeutic efficacy was determined in vitro and in vivo using murine (4 T1) and human triple-negative breast cancer cells (MDA-MB-231), and 4 T1-Luc-tumor bearing mouse models. The results have shown highly efficient in inducing apoptosis, cell cycle arrest (G2/M) phase, significant cytotoxicity, mitochondrial membrane depolarization, ROS generation, and photothermal effect leading to a higher proportion of cell death [59].

### Sonodynamic therapy

The therapy relies on the utilization of ultrasound waves to target and eliminate tumour cells by inducing an excess generation of oxygen reactive species through the Stimulation of sensitizers. Sonosensitizers can be categorized into two types: organic sonosensitizers and inorganic sonosensitizers. Examples of organic sonosensitizers encompass porphyrin and diisocyanate phthalate. Meanwhile, inorganic sonosensitizers include mesoporous silica nanoparticles and nano-titanium dioxide. These sonosensitizers promote the generation of ROS upon exposure to ultrasound waves, leading to the selective destruction of tumour cells [60, 61]. In an investigation conducted by Xiao Han et al., designed a new sonosensitizer which showed excellent performance in inhibiting cancer cells and in simultaneously suppressing the migration and invasion of cancer cells. In vitro and in vivo experiments showed a sonosensitizer could generate higher levels of reactive oxygen species thereby resulting in better anti-cancer effects [62].

### Gene therapy

This Approach involves introducing genetic material into target cells using a vector, followed by the modification, addition, or suppression of specific genes. The aim is to pinpointly target tumor cells While diminishing the impact on normal cells. Two commonly used methods in gene therapy are CRISPR and RNA-based approaches (e.g., miRNA and siRNA). CRISPR, along with CRISPR-associated protein 9 (CRISPR-Cas9), is a potent gene editing application that can efficiently knock out both cellular and viral oncogenes, enabling precise gene modifications in tumor cells. In a research work carried out by Jialong Fan et al., developed a combinational nanosystem for efficient TNBC therapy by programming tumor microenvironment and improving drug penetration ability. First, a hybrid membrane (HM) camouflaged poly lactic-co-glycolic acid (PLGA) nanosystem was developed for tumor-targeted delivery of capsaicin (Cap) to improve the tumor hypoxic microenvironment via dilating tumor blood vessels with oxygenation and promote the accumulation of nano-drugs in the tumor. The results were shown better results than the chemotherapy [63].

### Nano medicine

Various nanoparticle Preparations such as polymeric NPs, micelles, fullerenes, nanotubes, and lipid-based formulations (e.g., solid lipid nanocarriers, liposomes, and nano-emulsions), have demonstrated the ability to penetrate the leaky vasculature associated with tumours. The Tumour surroundings in hormone-receptor-negative breast tumours are complex, featuring heterogeneity in tumour endothelial cells, dense collagen-rich tumour tissue, extra-tumour matrix, and high intra-tumour pressure, which can impede the passive diffusion of nanocarriers. Nonetheless, some investigations have successfully utilized passive targeting through the enhanced permeability and retention effect to deliver drugs to the tumour site. These elements can affect the penetration of carriers based on particles and the buildup of drug delivery systems at the tumour location. Therefore, having a comprehensive understanding of the physiological characteristics of nanomedicine is essential for achieving effective delivery through both non-selective and selective targeting tactics [64].

To enhance the deposition of nanomedicine in specific regions, like ischemic tissue, tumour regions, or inflamed areas, formulation and delivery strategies are employed. Drug release mechanisms can be activated by factors like pH, redox potential, enzyme presence, and temperature. Active targeting approaches involve addressing the tumour microenvironment, including the vessel and extracellular matrix, using monoclonal antibodies, pH-sensitive drug carriers, non-platinum metal complexes, and magnetic nanoparticles (MNPs). These strategies not only fight the spreading condition, and enable imaging of TNBC but also reduce off-target toxicity, improve drug uptake, and enhance drug deposition in the cancerous growth tissues [65, 66].

## Advanced therapeutic approaches for triple-negative breast cancer

The presence of tumour heterogeneity offers significant hurdles in their diagnosis and medical care. The various phenotypes of cells within the tumour and the complexities of the tumour surroundings make it challenging to determine the most effective therapy. Often, a single treatment may not be enough to achieve optimal results. A major obstacle in chemotherapy is ensuring targeted drug delivery and accumulation at the precise spot. To deal with this, several precision medicine agents have been formulated. These agents facilitate targeted drug delivery, enhancing accumulation of the drug within the neoplasm. They exert their anti-tumour effects by directly engaging with cancer cells or modulating signalling pathways involved in tumours advancement and spread. These substances enhance the efficacy of BC therapy and may also synergize with other chemotherapeutic agents. These substances enhance these agents improve the efficacy of BC therapy and may also synergize with other chemotherapeutic agents [67–72]. Various targeted nanocarriers in different clinical phases were discussed in Table 2 for the effective recovery from TNBC. The nanocarriers were classified as Polymeric Nanoparticles (Polymeric nanoparticles, dendrimers, Carbon nanomaterials), Liposomes (Liposomes, Lipid-based drug delivery), Inorganic Nanoparticles (Inorganic nanoparticles), Nucleic Acid–Based Therapeutics and Exosomes.

**Table 2:** Various ongoing clinical trials with nano carriers at different clinical phases

Nanocarrier system	Therapeutic agents	ClinicalTrails.gov identifier No.	Therapeutic indication	Clinical trial phase
Albumin-stabilized NPs	PTX, Gemcitabine & Bevacizumab	NCT00662129	Metastatic Breast Cancer	Phase 2
	PTX, & Ixabepilone	NCT00785291	TNBC	Phase 3
	Carboplatin, PTX, & Bevacizumab	NCT00654836	Breast Cancer	Phase 2
	Carboplatin & PTX	NCT01525966	TNBC	Phase 2
Albumin-bound NPs	PTX, Epirubicin & Cyclophosphamide	NCT03799679	TNBC	Phase 4
	PTX & Carboplatin	NCT03799692	Luminal B/HER-2 Negative Breast Cancer	Phase 4
	PTX & Cyclophosphamide	NCT00629499	Breast Cancer	Phase 2
Albumin-stabilized NPs	Pertuzumab, Trastuzumab & PTX	NCT01730833	HER-2 Positive Breast Cancer	Phase 2
Albumin-stabilized NPs	PTX	NCT00609791	Metastatic mammary Cancer	Phase 2
Albumin-bound NPs	Pembrolizumab (Pbr)/PTX	NCT03289819	TNBC	Phase 2
Albumin-bound NPs	PTX & carboplatin	NCT04137653	TNBC	Phase 3
Albumin-bound NPs	PTX & Phenelzine Sulfate	NCT03505528	Metastatic mammary Cancer	Phase 1
Albumin-bound NPs	PTX Carboplatin, Trastuzumab/Bevacizumab	NCT00618657	HER-2 Positive & HER-2 Negative Breast Cancer	Phase 2
CdS/ZnS core–shell type quantum dots	Veldoreotide	NCT04138342	Breast Cancer	Phase 1
Lyso-thermosensitive liposomal DOX (ThermoDox)	ThermoDox in combination with Microwave Hyperthermia	NCT00826085	Breast Cancer	Phase 1
Liposome	Mitoxantrone Hydrochloride	NCT02596373	Metastatic mammary Cancer	Phase 2
Liposome	PTX	NCT01994031	Breast Cancer	Phase 4

Clinical translation of nanocarriers for the treatment of TNBC: Primarily ensuring the biocompatibility and safety of nanocarriers is important, requiring extensive preclinical toxicity studies to address concerns about immunogenicity and cytotoxicity. Secondarily, achieving specificity in targeting BSCS while minimizing off-target effects poses a significant hurdle. Nanocarriers must be engineered with precise targeting ligands or surface modifications to enhance tumor cell uptake and reduce systemic toxicity. Additionally, the heterogeneity of TNBC tumors, including variations in stem cell populations, complicates treatment strategies. Furthermore, BSCS exhibit inherent resistance to conventional therapies, necessitating the development of nanocarriers capable of overcoming drug resistance mechanisms. Additionally, transformation of preclinical studies to clinical trials involves navigating complex regulatory pathways and demonstrating safety, efficacy, and scalability.

### Nanomedicine for targeting BSCS in TNBC

The field of cancer was transformed by nanomedicine, offering innovative solutions for diagnosis and targeted drug delivery. Nanoparticles have become vital in delivering chemotherapy agents with improved effectiveness and decreased toxicity, surpassing limitations associated with traditional treatments. Nanocarriers possess the unique ability to traverse cell membranes and other barriers, enhancing drug diffusion and transfer [73–75]. However, the success of nanomedicine depends on stability, circulation time, and biocompatibility [76, 77].

Various forms of nanomedicine, such as drug-loaded nanoparticles, micelles, carbon nanotubes, solid lipid nanoparticles (SLNs), self-emulsifying drug delivery systems (SEDDS), nanostructured lipid carriers (NLCs), drug-antibody conjugates, and liposomes, have the ability to enter biological membranes and dispense drugs directly into cells (Fig. 2). The particle size, shape, and chemical characteristics of nanoparticles have a pivotal impact on their cellular absorption, distribution throughout the body, and the processes of elimination. Numerous nanoparticle-based chemotherapeutic delivery platforms are presently undergoing clinical assessments for treating various types of Breast cancer including TNBC. In this section we discuss various applications of nanomedicines in TNBC treatment [78–80].

A recent study focused on creating a polymeric nanoparticle (NP) system for the sequential dispensation of therapeutic substances in TNBC. The researchers incorporated an epidermal growth factor receptor inhibitor, erlotinib (Ei), and doxorubicin (Dox) into this innovative NP system. Their goal was to enhance the therapeutic effect by achieving Effective encapsulation capability and regulated release patterns. The polymeric NP system demonstrated promising results, showing improved drug delivery and therapeutic outcomes [81, 82].

In another study focused on creating a polymeric nanoparticle (NP) system for the sequential dispensation of therapeutic substances in TNBC. The researchers incorporated an epidermal growth factor receptor inhibitor, erlotinib (Ei), and doxorubicin (Dox) into this innovative NP system. Their goal was to enhance the therapeutic effect by achieving Effective encapsulation capability and regulated release patterns. The polymeric NP system demonstrated promising results, showing improved drug delivery and therapeutic outcomes [81, 82].

The NPs exhibited excellent cellular uptake, leading to enhanced cytotoxicity against TNBC cells. Moreover, the slow release of the encapsulated drugs from the NPs provided prolonged drug exposure and sustained efficacy. These results validate the possibility of NPs in delivering drugs specifically to TNBC tumours. The developed polymeric NP system offers a promising approach for the Progressive administration of EGFR inhibitors and doxorubicin, resulting in Improved healing outcomes. The observed improvements in cellular uptake, cytotoxicity, and tumour size reduction highlight the potential of NPs as carriers for TNBC treatment. Further research and development in nanoparticle delivery show significant promise in advancing therapy options and Results for individuals, with triple-negative breast cancer. In another study researchers investigated the potential of protein albumin-stabilized nanoparticles (NPs) loaded with gemcitabine and paclitaxel (PTX) and with bevacizumab monoclonal antibody to inhibit cancer expansion in breast cancer patients. The investigation aims to enhance the therapeutic efficacy of gemcitabine and PTX by encapsulating them within albumin-stabilized NPs. These NPs were further modified with bevacizumab mAb to target specific molecules involved in tumour angiogenesis. The goal was to suppress tumour growth and improve treatment outcomes in breast cancer patients [83–87].

Encouraging findings emerged from the phase II clinical study, indicating favourable results. The use of albumin-stabilized nanoparticles (NPs) loaded with gemcitabine and PTX, combined with bevacizumab functionalization, led to a notable decrease in tumour growth. This innovative method shows promise in enhancing breast cancer management by providing a targeted and effective drug delivery system. Additional research and medical investigations are necessary to validate these results and optimize the application of gemcitabine and bevacizumab-loaded nanoparticles in breast cancer therapy. With a focus on pioneering approaches like nanoparticle-based drug delivery, scientists are working to improve the therapeutic advantages and overall outlook for breast cancer patients with a focus on pioneering approaches like nanoparticle-based drug delivery, scientists are working to improve the therapeutic advantages and overall outlook for breast cancer patients [88–90]. Specific molecular targets and their specific action to eliminate BSCS were discussed in Table 3 as follows.

**Table 3:** Molecular Targets to eliminate cancer and BSCS

Molecular targets	Scientific targets	References
**Surface antigens**		
CD44	Necessary in Cellular Interactions, Attachment, and Movement	[91]
CD133	Suppression of CD133 Induces BCSC Exclusion and Inhibits Tumour Growth	[92]
EpCAM	BCSC Elimination Achieved through SiRNA-Mediated Inhibition of EpCAM	[93]
**Renewal signalling pathways**		
Wnt/β-catenin pathway		
Fz receptor	Inhibit Wnt Pathway in BCSCs by Monoclonal Antibody Targeting Frizzled Receptor	[94]
β-catenin	Selective Removal of Breast CSC and Non-Breast CSC via Wnt Pathway Inhibition with β-catenin (CWP232228)	[95]
Axin 2	Decrease in expression of Wnt/β-catenin Pathway and Loss of β-catenin Function through Small Molecule Inhibition of Axin2	[96]
**Signalling pathway-notch**		
γ- secretase	Suppression of Notch Signals in tumour stem cells, Mammosphere and Colony Formation via Inhibition of enzyme MRK-003-Mediated γ-Secretase	[97]
MAML	Inhibition of Notch Signaling through “ANTP/DN MAML Fusion Protein” Targeting “Notch Nuclear Co-activator MAML1”	[98]
“Delta-like 4 ligand” (DLL4)	Anticancer Activity through suppression of Notch Signaling Pathway via Monoclonal Antibodies or Small Molecule Inhibitors Targeting DLL4	[99]
**Pathway hedgehog (Hh)**		
Smo	Elimination of “BCSCs” and Prevention of Drug Resistance in “MCF-7/ADR Cells” through the Use of Smo Antagonist (Cyclopamine)	[100]
Glioma-associated oncogene (Gli)	Silencing of “Hedgehog Signal” through Effective suppression of Gli with Small Molecule Inhibitor	[101]
TGF- β	Elimination of BCSCs Achieved through Inhibition of TGF-β Signaling	[102]
Bmi-1	Inhibition of Tumour Proliferation and Metastatic Spread in Breast Cancer through Bmi-1 Silencing with SiRNA	[103, 104]
**IL-6/JAK/STAT3 pathway**		
STAT3	Elimination of “BCSCs” and Prevention of Tumour Metastasis through suppression of STAT3	[104]
**Apoptotic/antiapoptotic proteins**		
PTEN/PI3/Akt axis	Total elimination of the tumour Achieved through Inhibition of AKT and Suppression of Mammosphere Formation	[105]
m-TOR	Enhanced Sensitivity of BCSCs to Radiation through Interfering mTOR with Rapamycin	[106]
Bcl2	Improved Chemosensitivity of BCSCs Achieved by Bcl2 Silencing with SiRNA	[107]
Fatty acid synthase (FAS)	Stimulation of Apoptotic Pathways in BCSCs through Modulation of enzyme (FAS) Expression and Suppression of Lipogenesis	[108]
Death receptor (DR-5)	Cytotoxic Effects on BCSCs Achieved through the Use of Anti-DR5 Antibody	[109]
**Autophagy proteins**		
Beclin1	Inhibition of Autophagy in BCSCs through Depletion of Beclin1	[110]
**Biological catalysts and transporters**		
Hexose kinases (HK)	Tumour Growth Inhibition in Breast Cancers through Impaired Glucose Metabolism in BCSCs via HK Inhibition by Metformin	[111]
Glucose transporters (GLUT)	Enhanced Radiosensitivity of Breast Cancer Cells through Inhibition of GLUT1 Transporter	[112]
**Microenvironment**		
Hypoxia inducible factor1a (HIF1a)	Chemo sensitization of tumour stem cells Achieved through Restraint of “HIF1α”, a Promoter of BCSCs Growth	[113]
Carbonic anhydrase-IX (CAIX)	Elimination of BCSCs through Inhibition of CAIX Using a Novel Small-Molecule Inhibitor	[114]
CXCR1	Effective Elimination of BCSCs Achieved through Inhibition of CXCR1	[115]

### Polymeric nanocarriers

Protein-based polymeric micelles have gained recognition as efficient drug carriers, offering advantages such as significant drug loading effectiveness, prolonged drug release, and compatibility with biological systems, and cellular penetration. In a study, gelatin, a hydrophilic protein, was modified into an amphiphilic form by attaching it to oleylamine using genipin as a cross-linker. The resulting CT-GOC (Gelatin-Oleylamine Conjugate) nanocarriers were investigated for their efficacy in delivering drugs to TNBC-type cells (MDA-MB-231). The nanocarriers were found to be effectively internalized by the cancer cells, leading to cell cycle arrest at the G2/M phase and prompting cell death through programmed processes. These observations imply the potential of the GOC micelles as therapeutic transporters for innovative agents in TNBC chemotherapy. However, additional investigation and medical trials are required to validate their capability and protection before their use in clinical applications*.*

In a research investigation, the researchers aimed to address the challenges associated with the drug paclitaxel, including its limited dissolvability, reduced absorbability, and significant whole-body toxicity. To overcome these limitations, they developed stable micelles capable of effectively binding to paclitaxel and improving its delivery. The micelles were created using poly (ethylene glycol)-block-dendritic polylysine, and the primary amines of the polylysine were reacted with phenethyl isothiocyanate (PEITC), a hydrophobic anticancer agent. This led to the formation of paclitaxel-loaded micelles known as PEG-Gx-PEITC third-generation micelles (PEG-G3-PEITC/PTX). In the study, it was observed that the developed PEG-Gx-PEITC/PTX micelles displayed favourable pharmacokinetic properties. They exhibited a longer residency in the bloodstream due to slowed blood clearance. Moreover, the micelles demonstrated increased accumulation in neoplastic tissues, resulting in enhanced therapeutic efficacy. The improved accumulation of the micelles in tumour tissues translated into more favourable in vivo therapeutic outcomes in both subcutaneous and orthotopic human breast cancer xenograft models.

The study underscores the promise of PEG-Gx-PEITC/PTX micelles as an effective drug delivery platform for paclitaxel. Their improved pharmacokinetic properties and enhanced tumour accumulation offer a potential solution to overcome paclitaxel's limitations, thereby enhancing its therapeutic efficacy in breast cancer treatment. However, additional research and rigorous testing are needed to confirm, these results and establish the safety and effectiveness of these micelles in clinical applications [116, 117]. In a recent study, polyethylene glycol (PEG) nanoparticles (NPs) were developed to encapsulate Decitabine (DAC), a DNA hypermethylation inhibitor. The main objective of this formulation was to improve the response to chemotherapy and overcome drug resistance in breast cancer stem cells and MDA-MB-231 cells. The researchers utilized self-assembled block copolymer-based nanocarriers composed of poly (d, L-lactide-co-glycolide) and hyaluronic acid for delivering a synergized combination of Salinomycin and paclitaxel (PTX) to BCSCs. The incorporation of hyaluronic acid facilitated drug uptake through CD44 + receptors, leading to increase in vitro activity. The study findings suggest the potential of these PEG NPs in targeting BCSCs and enhancing the efficacy of chemotherapy, offering promising implications for breast cancer treatment. Further research and investigations are needed to authenticate these results and assess the clinical applicability of this novel drug delivery approach in their study, Li et al. investigated the co-delivery of Dasatinib and mTHPC (a photosensitizer) using a poly (lactic-co-glycolic acid) (PLGA) nano-core. The combination of these two agents demonstrated synergistic cytotoxicity in both metastatic MDA-MB-231 cells and non-metastatic MCF-7 cells, achieved through a photo-activated oxidative stress mechanism. The photosensitizer played a crucial role in inhibiting SRC, leading to apoptosis in metastatic cancer cells. This photodynamic killing approach, utilizing nanomedicine, presents a promising and innovative strategy for combating triple-negative breast cancer. The findings from this study offer potential implications for the development of effective and precision treatments for TNBC treatment. Further investigations are necessary to explore the clinical applicability and safety of this approach in mammary carcinoma patients in this research, the researchers utilized polymeric micelles, which are supramolecular delivery systems made of amphiphilic block copolymers. These micelles have a semi-solid hydrophobic core composed of biodegradable polymers like poly(L-lactide), poly(β-caprolactone), or PLGA. The micelles possess a size ranging from 10 to 100 nm, making them an excellent platform for delivering water-insoluble chemotherapies. Their unique structure and properties enable efficient and specific drug conveyance, offering potential advantages in the treatment of various diseases, including cancer [118, 119].

In conclusion, the research showcases the capability of PEG nanoparticles encapsulating DAC and polymeric micelles as innovative approaches for drug delivery in breast cancer stem cells (BCSCs) and triple-negative breast cancer cells. The incorporation of hyaluronic acid and the use of a photosensitizer exhibited promising results, indicating improved therapeutic effects and the possibility of overcoming medication insensitivity. These findings contribute to the growing field of nanomedicine and photodynamic approaches in the treatment of TNBC. However, additional research is essential to validate and optimize these strategies for potential clinical use, ensuring their safety and efficacy. However, additional research is essential to validate and optimize these strategies for potential clinical use, ensuring their safety and efficacy [120–123].

### Inorganic nanoparticles

A noteworthy study exploring the potential of silver nanoparticles (AgNPs) for drug delivery and diagnostics in TNBC. Their findings revealed that AgNPs displayed significant cytotoxicity specifically towards TNBC cells, while non-malignant breast epithelial cells remained unharmed at the doses administered. Intriguingly, both TNBC cells and healthy mammary epithelial cells exhibited similar sensitivity to silver cations (Ag^+^), suggesting that the unique cytotoxicity towards TNBC cells is primarily attributed to the nanoparticle formulation rather than the release of Ag^+^ alone. The researchers investigated the impact of silver nanoparticles (AgNPs) on TNBC cells and healthy mammary epithelial cells. They observed that AgNPs specifically induced oxidase stress and depleted antioxidants in TNBC cells, without causing similar effects in non-malignant breast epithelial cells. Moreover, AgNPs caused significant DNA damage in TNBC tumour nodules in a three-dimensional (3D) cell culture model, while maintaining the integrity of normal breast acini and not inducing DNA damage or apoptosis. The cytotoxicity of AgNPs was attributed to the release of silver cations (Ag^+^). The study further revealed that both TNBC cells (MDA-MB-231 and SUM159) and non-malignant breast epithelial cells (MCF-10A and iMEC) were sensitive to Ag^+^ exposure, but TNBC cells showed faster degradation of AgNPs compared to non-malignant cells. These findings shed light on the selective cytotoxicity of AgNPs towards TNBC cells and highlight their potential in targeted therapy and diagnostic applications for TNBC.

The findings from this study offer valuable insights into the potential applications of AgNPs in the context of triple-negative breast cancer. Their selective cytotoxicity towards TNBC cells and the potential to stimulate DNA damage in tumour nodules suggest that AgNPs could serve as a possible model for targeted therapy. Moreover, the differential degradation rates observed between TNBC cells and non-malignant breast epithelial cells open up possibilities for developing more specific and efficient drug delivery strategies.

Despite these promising findings, it is essential to conduct further research to gain a comprehensive insight into the core mechanisms and to address potential safety concerns. Optimizing the use of AgNPs in clinical settings requires careful consideration of their interactions with normal cells and tissues, potential long-term effects, and potential accumulation in the body.

As the field of nanomedicine continues to advance, AgNPs hold great potential as a versatile tool for drug delivery and diagnostics in TNBC and other cancer types. Continued research and development in this area will pave the way for innovative and effective treatment approaches in the future [124]. In other study, A. Mohammed Siddiq et al. utilized an innovative approach to synthesize gold nanoparticles (AuNPs) using an aqueous extract of neem fruit (Azadirachta indica) as a reducing agent. As the field of nanomedicine continues to advance, AgNPs hold great potential as a versatile tool for drug delivery and diagnostics in TNBC and other cancer types. Continued research and development in this area will pave the way for innovative and effective treatment approaches in the future [124].

The main goal of the study was to explore the potential of AuNPs as novel anticancer agents specifically targeting triple-negative breast cancer cells. The researchers aimed to evaluate the stability and effectiveness of these nanomaterials for potential use in TNBC treatment.

This novel approach holds promise for advancing the development of targeted anticancer therapies for TNBC. By utilizing neem fruit extract the researchers have taken significant steps towards the realization of effective and stable AuNPs with potential applications in TNBC treatment. However, further research and investigation are necessary to fully understand the mechanisms involved and to assess their safety and efficacy in clinical settings. In their study, the researchers conducted in vitro cytotoxicity tests to assess the effects of the synthesized AuNPs on both the TNBC cell line MDA-MB-231 and normal NIH3T3 cells. The outcomes showed that the AuNPs effectively hindered the proliferation of TNBC cells directly. Additionally, the AuNPs induced a process called tumour cell autophagy, which contributed to an increase in cellular apoptosis or programmed cell death.

These findings indicate the potential of the synthesized AuNPs as a targeted treatment strategy for triple-negative breast cancer. By selectively inhibiting TNBC cell growth and promoting tumour cell autophagy leading to apoptosis, these AuNPs show promise as a novel anticancer agent. However, further studies are necessary to validate these observations and explore their full therapeutic potential in combating TNBC. Additionally, rigorous investigations on safety and biocompatibility will be essential before their translation into clinical applications.

The study on the synthesized gold nanoparticles (AuNPs) indicates their potential as novel anticancer agents for treating triple-negative breast cancer (TNBC). The AuNPs demonstrated the capability to impede TNBC cell growth and induce autophagy, ultimately leading to cellular apoptosis, which highlights their promising therapeutic applications. However, further investigation is required to comprehensively understand the underlying mechanisms of their anticancer effects and to optimize the AuNPs for potential clinical use in TNBC treatment. Further investigations are essential to explore their interactions with TNBC cells, affected signalling pathways, and impact on cellular processes. Additionally, optimizing the formulation, dosing, and stability of the AuNPs is crucial for advancing their potential as effective and safe anticancer agents in TNBC treatment. Further studies on their pharmacokinetics, biodistribution, and potential side effects will help ensure their safety and efficacy in vivo. Moreover, exploring the possibility of combining the AuNPs in combination with other treatment approaches, such as chemotherapy or immunotherapy, could enhance their therapeutic outcomes. Overall, while the AuNPs show promise as a potential treatment option for TNBC, additional research is necessary to move closer to their clinical application and improve the outlook for TNBC patients. Overall, while the AuNPs show promise as a potential treatment option for TNBC, additional research is necessary to move closer to their clinical application and improve the outlook for TNBC patients.

In an analysis led by Ghosh et al., synthesized novel gold nanoparticles (AuNPs) and silver nanoparticles (AgNPs) using a unique method referred to as GSTE (the exact details of GSTE were not provided in the given information). The researchers observed that both AgNPs and AuNPs demonstrated enhanced cytotoxicity against MCF-7 cells, a cell line representative of breast cancer cells. These findings suggest the potential of AgNPs and AuNPs as promising candidates for further investigation in breast cancer treatment. Yet, additional research is essential to fathom the mechanisms underlying their cytotoxic effects and to optimize their use for potential clinical applications*.*

The increased cytotoxicity observed indicates the promising potential of AgNPs and AuNPs as effective agents for targeting and inhibiting the growth of MCF-7 cells. However, additional research is required to comprehensively grasp the core mechanisms and optimize the synthesis and application of these nanoparticles for safe and effective clinical utilization in breast cancer treatment. In a distinct investigation, researchers examined the impact of Bismuth lipophilic nanoparticles (BisBAL NPs) on the MCF-7 cell line. The outcomes revealed a substantial induction of cell apoptosis by the BisBAL NPs. These observations imply the promising capability of BisBAL NPs as a viable approach to promote apoptosis in MCF-7 cells. The analysis executed highlights the eco-friendly nature of nanoparticles (NPs) as therapeutic conveyance system for potential treatment of TNBC. In a specific study, the efficacy of a Bi (III) hydrazine complex was assessed against MCF-7 cell lines. The in vitro evaluation results demonstrated the effectiveness of the Bi (III) hydrazine complex against “MCF-7 cells”. These findings hold promise for the potential use of NPs, like the Bi (III) hydrazine complex, as effective treatments for TNBC. However, additional research is necessary to acquire a more profound insight into the fundamental, mechanisms and to optimize their application in TNBC therapy. However, additional research is necessary to acquire a more profound insight into the fundamental, to gain a deeper understanding of the underlying mechanisms and to optimize their application in TNBC therapy [87, 125–129].

Combination drug therapy is a valuable approach utilized in cancer treatment to counteract drug resistance and enhance treatment efficacy. In a recent study led by Chandrasekaran Karthikeyan et al., researchers developed inorganic nanoparticles (NPs) with potential applications in anticancer therapy. Specifically, they created nanoparticles using tin dioxide (SnO2) and sodium alginate (SA), resulting in SnO2-based NPs known as SASnO2. Biomedical investigations revealed that SASnO2 NPs exhibited enhanced interaction with biological membranes and improved internalization (cell uptake) by tumour cells. This led to enhanced anticancer effects, primarily attributed to the larger surface area of “SASnO2 NPs”. These findings indicate the promising potential of SASnO2 NPs as nanomedicines for cancer treatment. Further research is necessary to explore their efficacy and optimize their application in clinical settings. Further research is necessary to explore their efficacy and optimize their application in clinical settings.

In a study researchers synthesized nanoparticles prepared by zinc oxide (ZnO-NPs) employing an environmentally friendly technique, employing Aloe barbadensis leaf extract as a stabilizing and capping agent. The capabilities of these ZnO-NPs as a drug delivery system were investigated by encapsulating them with “doxorubicin” (“DOX’). The efficacy of the DOX nanoparticles was evaluated against the “MDA-MB-231 cell line”, which represents TNBC. The study assessed the antiproliferative potential of the nanoparticles using the MTT assay. The study findings indicated that the biogenically synthesized PEGylated ZnO-NPs displayed improved therapeutic efficacy for treating TNBC. Notably, the doxorubicin-loaded PEGylated ZnO-NPs demonstrated the highest cytotoxicity among the tested formulations, exhibiting potent anticancer activity at a low concentration threshold.

The results emphasize the promising role of PEGylated-ZnO-NPs as a potential drug delivery approach to enhance therapeutic approaches to, triple-negative breast cancer. Further studies are needed to delve into the mechanisms engaged and, optimize the formulation to ensure its potential effectiveness and safety for future clinical use in TNBC treatment “Silica nanoparticles” (NPs) has surfaced as valuable tools for drug delivery and tumour imaging, with particular applications in breast cancer (BC). Specifically, targeted silica NPs loaded with fluorescent “cRGDY-PEG-Cy5.5-C” dots show promise for lymph node mapping in BC diagnosis and therapy. This targeted approach allows for sentinel lymph node mapping before surgery, enabling early detection of BC. The ongoing phase I/II clinical investigation is advancing towards clinical validation and implementation, showcasing its potential in BC management. The ongoing phase I/II clinical investigation is advancing towards clinical validation and implementation, showcasing its potential in BC management [87, 130–133].

### Liposomes

These are spherical vesicles with a unique closed composition, consisting of an aqueous solution enclosed by a lipid bilayer membrane. First conceptualized in 1964, liposomes marked a significant breakthrough in their development and study. Since then, they have garnered significant attention and have been widely researched or diverse uses, including pharmaceutical transport, gene therapy, and cosmetic formulations. These adaptable lipid-based structures remain a subject of active investigation and show immense promise in multiple fields of science and medicine. In 1971, a ground breaking study marked a significant milestone in the utilization of liposomes, paving the way for extensive research and exploration of these versatile drug delivery systems. Subsequently, liposomes have undergone comprehensive investigation and showcased their applicability in diverse areas of medicine and biotechnology. The pioneering work of Gregoriadis et al. played a crucial role in in laying the foundation for the growth and advancement of liposomal technologies. Liposomes are globular vesicles typically spanning in size from 50 to 100 nm. They possess the unique ability to enclose hydrophobic drugs within their bilayer of lipid and water loving drugs, genes, and siRNA within their aqueous core. Liposomes offer several advantages, such as decreased systemic toxicity prolonged duration of circulation, compatibility with biological systems, and limited uptake by the reticuloendothelial system, leading to slower elimination rates. These favourable attributes make liposomes an attractive choice for drug delivery systems with potential applications in various biomedical fields. Limited uptake by the reticuloendothelial system, leading to slower elimination rates. These favourable attributes make liposomes an attractive choice for drug delivery systems with potential applications in various biomedical fields [134, 135].

In their study, Alexandra Sneider et al. aimed to overcome drug solubility challenges by utilizing Liposomes as conveyors for drug transport. Liposomes are nanostructures that can enclose both lipophilic and hydrophilic drugs, and they have already been authorized for clinical use in cancer treatment. The researchers focused on liposomes loaded with benzoporphyrin derivative (BPD) and coated with polyethylene glycol (PEG), which were further conjugated with folate. This design aimed to enable precision delivery of the liposomes to tumour cells, particularly those expressing high levels of folate receptors, such as TNBC cells. In vitro experiments demonstrated the liposomes' efficient delivery of “BPD to MDA-MB-231” cells and their ability to trigger cell death through photodynamic therapy in both monolayer and 3D cell culture models. These findings indicate the potential of folate-targeted PEG-coated liposomes as effective vehicles for drug delivery and PDT-based treatment in TNBC. These findings indicate the potential of folate-targeted PEG-coated liposomes as effective vehicles for drug delivery and PDT-based treatment in TNBC [136, 137].

In a study developed lipid-based carriers for precision delivery of cisplatin to neoplasms. These conveyors have a nanometre size, enabling them to specifically gather in tumours. The carriers were engineered to possess specific properties within the tumour interstitium. Firstly, they demonstrated interstitial drug release, enabling deeper penetration of cisplatin within the tumour. Secondly, they exhibited attachment to the tumour’s “extracellular matrix” in the intratumoral /interstitial space, without being internalized by the cells. This adhesion resulted in delayed clearance of the carriers from the tumour, extending the duration of cancer cell exposure to the released cisplatin. These findings highlight the capability of the “lipid-based carriers” for targeted and effective drug delivery to tumours [134–136].

In a recent study, researchers investigated “lipid nanoparticles” with distinct properties—discharge and attachment, for targeted cisplatin delivery to tumours. The most effective nanoparticles were those with attributes encompassing both release and adhesion, followed by nanoparticles with only release or adhesion capabilities. In vivo experiments demonstrated that cisplatin-loaded nanoparticles possessing releasing and/or adhering characteristics markedly impeded the proliferation of spontaneous TNBC metastases, outperforming conventional liposomal cisplatin. The effectiveness of various permutations of release and adhesion characteristics in vivo aligned with the trends observed in spheroid models. These findings underscore the potential of lipid nanoparticles with specific properties for improved and targeted drug delivery in TNBC treatment [137].

The findings of the research underscore the promising potential of lipid-based nanoparticles with selective release and adhesion properties as an effective approach for targeted cisplatin delivery and inhibiting TNBC metastases. In their research, Parvani et al. developed liposomes specifically targeted to BCSCs and the cellular membrane receptor nucleolin, which is recognized for its elevated expression in TNBC cells. The liposomes were engineered with F3 peptide modifications to enable precise recognition and binding to BCSCs and nucleolin on the cell surface [138, 139].

The F3 peptide-targeted liposomes exhibited a remarkable capability to selectively deliver therapeutic agents to TNBC cells, particularly the cancer stem cells accountable for initiating and promoting tumour growth. This targeted approach holds tremendous promise for enhancing the effectiveness of TNBC treatments by specifically addressing the challenging aspects of tumour heterogeneity and drug resistance. The development of F3 peptide-targeted liposomes represents a hopeful and advanced strategy for improving drug delivery and achieving better therapeutic outcomes in TNBC treatment. The development of F3 peptide-targeted liposomes represents a hopeful and advanced strategy for improving drug delivery and achieving better therapeutic outcomes in TNBC treatment [140, 141].

### Dendrimers

Dendrimers have garnered considerable attention as nanocarrier systems in cancer chemotherapy, mainly due to their capacity to utilize ligand or receptor-mediated endocytosis, offering multiple advantages over traditional therapies. These nanostructures are characterized by a precisely defined, consistent, and uniformly distributed extensive, typically ranging in diameter from 2 to 10 nm. Dendrimers possess distinct characteristics that render them well-suited and aimed at delivery of treatment and diagnostic substances. Their precise and controlled structure enables efficient loading and release of drugs, facilitating specific targeting to cancer cells. This targeted approach shows potential for enhancing treatment outcomes by enhancing drug potency and minimizing off-target effects [142–144].

By capitalizing on the unique characteristics of dendrimers, researchers are striving to enhance the administration of therapeutic and diagnostic substances in cancer treatment, leading to novel and effective personalized strategies. In another study a nanocarrier system utilizing Polyamidoamine (PAMAM) dendrimers to deliver doxorubicin (Dox) and lycopene (LCP). This system also incorporated anti-survivin siRNA, forming a complex termed DLP/siRNA. In vitro studies demonstrated increased apoptosis and enhanced uptake of tumor cells when employing this dendriplex formulation. Additionally, gel retardation assays confirmed the dendrimer's capability to shield siRNA from nuclease degradation. In a study conducted by Sergio Andrés Torres-Pérez et al., a straightforward method was utilized to prepare “PAMAM dendrimers loaded with methotrexate and d-glucose”. The researchers investigated these conjugates in “TNBC cell lines”, specifically “MDA-MB-231”. The survival of the conjugates was evaluated using the “MTT assay” in both the “TNBC cell line” and “non-cancerous HaCaT cells”. Additionally, uptake investigations indicated that glycosylation enhanced the internal uptake of “OS-PAMAM” conjugates in tumour cells by approximately two-fold compared to non-cancer cells [145–147].

The studies conducted demonstrate the versatility of PAMAM dendrimers as nanocarriers for precise delivery of anticancer drugs in the management of “triple-negative breast cancer”. By encapsulating chemotherapeutic drugs and protective agents within the dendrimer structures, these nanocarriers offer a favourable method to enhance treatment outcomes and reduce off-target effects. The studies conducted demonstrate the versatility of PAMAM dendrimers as nanocarriers for precise delivery of anticancer drugs in the management of TNBC. By encapsulating chemotherapeutic drugs and protective agents within the dendrimer structures, these nanocarriers offer a favourable method to enhance treatment outcomes and reduce off-target effects [146, 148, 149].

Poly (propylene imine) (PPI) dendrimers, based on propylene imine monomers branching, have emerged as promising candidates for drug administration and nucleic acid transport in breast cancer (BC) treatment. These dendrimers undergone extensive research and functionalization with ligands to enhance their targeting capabilities. An investigation carried out by Gupta et al. explored the potential of the PPI dendrimers of the fifth generation, conjugated with folic acid (FA), to specifically targeting folate receptors. This targeted delivery system demonstrated successful delivery of doxorubicin (DOX), a commonly used chemotherapeutic agent, to the intended sites in BC cells. These findings indicate the promising potential of FA-conjugated PPI dendrimers for effective drug delivery in BC treatment. The specific binding of “PPI dendrimers with FA” enables them to selectively targeting and bind to folate receptors, which often highly expressed in breast cancer (BC). This targeted strategy leads to increased accumulation of DOX in BC cells, enhancing its therapeutic effectiveness and minimizing off-target effects. The application of PPI dendrimers as drug carriers in BC shows potential for improving treatment outcomes and reducing the adverse effects typically associated with conventional chemotherapy. The application of PPI dendrimers as drug carriers in BC shows potential for improving treatment outcomes and reducing the adverse effects typically associated with conventional chemotherapy [148, 150, 151].

### Lipid-based drug delivery

They offer a promising approach to enhance drug delivery to the tumour tissues, minimize side effects, and overcome multidrug resistance in cancer therapy.

Lipid-based nanomedicines, composed of colloids with lipid materials, present a versatile and promising approach in tumour investigation for both therapy and detection purposes. These formulations allow for efficient encapsulation and protection of drug compounds, leading to enhanced targeting and controlled release within tumour tissues. This approach improves the efficiency of cancer treatments and reduces toxicity to normal tissues. Moreover, lipid-based nanomedicines have the potential to overcome drug resistance mechanisms, making them valuable tools in tackling multidrug resistance in cancer cells [152, 153].

Lipid-based nanomedicines show immense potential in advancing cancer therapeutics, offering innovative and personalized treatment options through optimized drug delivery. “Nanostructured lipid carriers” (NLC), “solid lipid nanoparticles” and “self-micro/nano emulsified drug delivery systems” are some of the lipid-based formulations that offer distinct advantages. These formulations are biodegradable, ensuring safe breakdown within the body, and demonstrate excellent biocompatibility, reducing the risk of adverse reactions. The utilization of lipid-based nanomedicines opens up new avenues for enhancing cancer treatments and tailoring therapies to individual patient needs [154, 155].

Moreover, lipid-based nanomedicines can be engineered to enhance their targeting abilities, enabling precise and selective delivery of medications to particular locations in the body. By incorporating targeting ligands or surface modifications, these formulations can interact specifically with tumour cells or particular tissues, thereby improving the effectiveness of treatments while minimizing off-target effects. This targeted approach holds promise for advancing cancer therapy and improving patient outcomes. The adaptability of lipid-based nanomedicines allows for the incorporation of diverse drugs, both hydrophobic and hydrophilic, providing a wide range of therapeutic possibilities. Additionally, their nanostructured composition ensures enhanced stability and controlled release profiles, optimizing drug delivery and maximizing treatment efficacy. The overall advantages of lipid-based nanomedicines, encompassing NLCs, SLNs, SMEDDS, SNEDDS, and other lipid-based systems, include biodegradability, biocompatibility, and the potential for targeted delivery. These attributes make them valuable assets in pharmaceutical research and development, offering promising solutions for improved drug delivery and patient care. These attributes make them valuable assets in pharmaceutical research and development, offering promising solutions for improved drug delivery and patient care [156, 157].

In a groundbreaking study where they introduced an innovative strategy for breast cancer therapy, employing a conjugate of “Tamoxifen” (“TAM”) and “Resveratrol” (“RES”) encapsulated within Vesicle-by-Vesicle nanoparticles. These nanoparticles were developed using lipid-mediated medication transport systems and “liquid crystalline nanoparticles” (LCNPs). To enhance their targeting capability, the LCNPs were covered with several tiers of cationic chitosan and anionic hyaluronic acid. This novel approach holds great promise for advancing breast cancer treatment by delivering a mixture of medicinal substances within a targeted and regulated approach. The therapeutic potential of “TAM/RES–LbL-LCNP’s” was evaluated through the MTT assay, revealing a notable decrease in cell proliferation upon nanoparticle treatment. Significantly, these liquid crystalline nanoparticles exhibited remarkable therapeutic effectiveness while maintaining a safe and biocompatible profile. Rigorous testing on human red blood cells demonstrated no observable toxicity or adverse reactions. Furthermore, in mice, no behavioural abnormalities were detected, affirming the suitability of these nanoparticles for biomedical purposes. These promising results indicate the capability of “TAM/RES–LbL-LCNP’s” as a valuable approach for breast cancer treatment. Further research and medical examinations are needed to confirm their efficacy and safety in human subjects. The findings from this research emphasize the promising potential of the lipid-based LbL-LCNPs as a secure and efficient approach for breast cancer treatment. By incorporating TAM and RES within these nanoparticles, along with their distinctive properties, targeted drug delivery can be achieved, potentially leading to enhanced treatment efficacy for individuals with breast cancer. Further research and clinical investigations will be essential to validate and optimize the application of these nanoparticles in breast cancer therapy. “Self-emulsified drug delivery systems” (SEDDS) provide a notable benefit in transporting extremely hydrophobic medications, as they greatly enhance solubility. An illustration of this advantage is demonstrated through the creation of self-emulsified drug delivery systems containing docetaxel (DTX), which were then assessed for their efficacy in cancerous cells in the breast [87, 158, 159].

The researchers developed a SEDDS preparation for enhanced solubility and delivery of DTX. Through *In-Vitro* experiments on breast cancer cells, the DTX-loaded SEDDS demonstrated enhanced efficacy in delivering the drug to the target cells. This approach shows potential for improving therapeutic outcomes and overcoming the obstacles related to administering highly lipophilic drugs. This approach shows potential for improving therapeutic outcomes and overcoming the obstacles related to administering highly lipophilic drugs [158].

### Carbon nanomaterials

These includes nanodiamonds, Nanotubes, nanofibers, Graphene and its derivatives, including graphene oxides, have shown significant potential as nanomaterials with various clinical applications for handling “Triple-negative breast cancer”. In another investigation they created “oxidized mesoporous carbon nanoparticles” (oMCNs) using a gentle oxidation technique. These oMCN’s possessed dimensions beneath 200 nm and superb aqueous dispersibility. Moreover, the researchers successfully loaded the drug RES within the cavities, achieving a high strong drug encapsulation efficiency of 24.8% (w/w). The study demonstrated that RES encapsulated in oMCNs demonstrated enhanced cytotoxicity against “MDA-MB-231” cells in a manner directly proportional to the dosage of the free drug. This highlights the potential of oMCNs as effective carriers for enhancing the therapeutic efficacy of RES and targeting TNBC cells. This highlights the potential of oMCNs as effective carriers for enhancing the therapeutic efficacy of RES and targeting TNBC cells [116, 160, 161].

In a study conducted by researchers, PEGGO nano-sheets loaded with SN38, a camptothecin analogue, demonstrated notable inhibitory effects on cell proliferation on various TNBC cells, including “MDA-MB-436”, “SK-BR-3”, “MDA-MB-231”, and “MCF-10A” cells, in in-vitro experiments. These nano-sheets also showed potential inhibitory effects In a study conducted by researchers, PEGGO nano-sheets loaded with SN38, a camptothecin analogue, demonstrated notable inhibitory effects on cell proliferation on various TNBC cells, including “MDA-MB-436”, “SK-BR-3”, “MDA-MB-231”, and “MCF-10A” cells, in in-vitro experiments. These nano-sheets also showed potential inhibitory effects on the invasion and metastasis of cancer cells. Additionally, in another study, quantum dots coated with veldoreotide, a somatostatin analogue, were utilized for tumour mapping and reducing tumour growth in breast cancer. Currently, this approach is in the phase I clinical trial stage. These innovative nanomedicine strategies hold promise for advancing TNBC treatment and cancer therapeutics in general. Overall, the utilization of PEGGO nano-sheets loaded with SN38 and veldoreotide-coated quantum dots shows promise in combating TNBC by exerting anti-proliferative effects, inhibiting cancer cell invasion, and potentially reducing tumour growth. Overall, the utilization of PEGGO nano-sheets loaded with SN38 and veldoreotide-coated quantum dots shows promise in combating TNBC by exerting anti-proliferative effects, inhibiting cancer cell invasion, and potentially reducing tumour growth [148, 162, 163].

### Nucleic acid-based therapeutics

Nanocarriers offer versatile delivery systems for a broad, array of remedial compounds, serving various applications in medicine. The nucleic acid delivery system has gained considerable attention as a potential method for managing diverse conditions, showing encouraging results in current research. Different isothermal amplification techniques have been created direct attention to malignant cells using therapeutic “Nucleic Acids”, with rolling circle amplification (RCA) being one notable example. Through chemical modifications and strategic integration with nano-sized entities, nucleic acids can be enhanced, providing distinct physical and chemical properties that advance tumour-targeted therapies. In a study led by Kaiyuan Xing et al., a bioinformatics approach using “single-cell RNA-seq” was proposed to ascertain “TNBC subtype-specific prognosis signatures” (TSPSigs). The analysis revealed that “TSPSigs” in two “TNBC” subtypes, “BL1” and “LAR”, were self-sufficient predictive determinant. Moreover, the expression of TSPSigs in TNBC cell lines showed significant associations with drug sensitivities. These findings contribute to the exploration of category correlated with molecular markers for TNBC detection and therapy, thereby facilitating the development of customized therapy plans for patients with different TNBC subtypes. Nanocarriers offer versatile delivery systems for a broad, array of remedial compounds, such as Nucleic Acids, serving various applications in medicine. The nucleic acid delivery system has gained considerable attention as a potential method for managing diverse conditions, showing encouraging results in current research. Different isothermal amplification techniques have been created direct attention to malignant cells using therapeutic Nucleic Acids, with rolling circle amplification (RCA) being one notable example. Through chemical modifications and strategic integration with nano-sized entities, nucleic acids can be enhanced, providing distinct physical and chemical properties that advance tumour-targeted therapies. In a study led by Kaiyuan Xing et al., a bioinformatics approach using “single-cell RNA-seq” was proposed to ascertain “TNBC subtype-specific prognosis signatures” (TSPSigs). The analysis revealed that “TSPSigs” in two “TNBC” subtypes, “BL1” and “LAR”, were self-sufficient predictive determinant. Moreover, the expression of TSPSigs in TNBC cell lines showed significant associations with drug sensitivities. These findings contribute to the exploration of category correlated with molecular markers for TNBC detection and therapy, thereby facilitating the development of customized therapy plans for patients with different TNBC subtypes [164, 165].

Clinical evidence has highlighted the significant challenge of drug resistance in breast cancer (BC) treatment, leading to higher mortality rates and cancer recurrence. The development of medication insensitivity is influenced due to alterations in tumour genetics cells. In the recent past, genetic manipulation techniques, notably the “Clustered Regularly Interspaced Short Palindromic Repeats” “CRISPR” and “Cas9” polypeptides, have evident as a promising approach to address drug resistance. The CRISPR/Cas9 technology allows for precise identification and targeting of specific genomic regions associated with drug resistance in BC. By reversing these genetic alterations, this technology shows promising potential for treating drug-resistant breast cancer and provides new therapeutic opportunities. By reversing these genetic alterations, this technology shows promising potential for treating drug-resistant breast cancer and provides new therapeutic opportunities [15, 166].

Numerous nanoparticulate systems have undergone investigation as potential carriers for CRISPR systems as therapy for “Triple-negative breast cancers” (TNBCs). “CRISPR/Cas9”, a DNA-editing technique, holds promise for correcting genetic errors with precision. This technique allows for targeted activation or deactivation of specific genes within cells. Positive findings have been witnessed in preclinical studies using animal models, such as mice, where faulty DNA was repaired, offering potential for treating genetic disorders. The CRISPR/Cas9 method involves using “single guide RNA” (“sgRNA”) to direct the “Cas9 nuclease” to matching segments of the genetic material. Once bound, Cas9 cleaves the DNA, initiating the repair process via double-stranded breaks (DSBs). The “CRISPR/Cas9” system signifies highly encouraging, advancement in cancer gene therapy. Its versatile applications in transcriptional regulation and gene editing offer precise control over gene expression and targeted modifications of the genetic material. Once bound, Cas9 cleaves the DNA, initiating the repair process via double-stranded breaks (DSBs). The “CRISPR/Cas9” system signifies highly encouraging, advancement in cancer gene therapy. Its versatile applications in transcriptional regulation and gene editing offer precise control over gene expression and targeted modifications of the genetic material. As a powerful tool, CRISPR/Cas9 holds great potential for therapeutic benefits in cancer treatment. The CRISPR/Cas9 technology has shown promising potential for treating various hereditary disorders, including breast cancer. Its versatility and high efficacy make it an attractive gene therapy tool in cancer treatment. However, delivering Cas9 and sgRNA to tumour site effectively continues to be an obstacle. To confront this, researchers have explored different approaches. For instance, studies by Frazer Sinclair et al. and Zhang et al. have focused on “PEG phospholipid-modified cationic lipid nanoparticles” (“PLNP”) to enhance the transfection efficiency of “CRISPR/Cas9” in tumours. These innovative delivery systems aim to improve the targeted delivery of CRISPR components, leading to efficient gene editing and therapeutic outcomes in mammary gland malignancy, specifically in “Triple-negative breast cancer” (TNBC). Investigators have formulated, PEG phospholipid-modified cationic lipid nanoparticles (PLNP) as an optimal distribution system for Cas9/sgRNA plasmids in cancer cells. This core–shell structure, referred to as PLNP/DNA, facilitates the encapsulation of “Cas9/sgPLK-1 plasmids”. In mouse models, the infusion of “Cas9/sgPLK-1 plasmids” led to significant inhibition of “Polo-like kinase-1 (PLK-1) protein” followed by decreased tumour expansion. This study demonstrates the versatility and effectiveness of utilizing the “CRISPR/Cas9” arrangement for delivery to target, both “*In-Vitro”* and “*In-Vivo”*, with improved efficacy [167–169].

### Exosomes

Exosomes, natural membranous vesicles, play crucial roles in numerous biological and pathological processes. These vesicles have gained attention for their potential in drug delivery, particularly in cancer treatment. With benefits like non-immunogenicity and extended circulation time, exosomes present promising advantages as pharmaceutical delivery platforms. Composed of broad spectrum of proteins and lipids naturally found in the human body, exosomes hold significant therapeutic potential. Composed of broad spectrum of proteins and lipids naturally found in the human body, exosomes hold significant therapeutic potential [170, 171].

In the context of mammary carcinoma and TNBC, the use of cytotoxic medications has the potential to eventually cause the emergence of treatment resistance. This resistance may be moved from resistant malignant cells to sensitive cells through exosomes, which carry miRNA and associated proteins. This process underscores the important function of exosome-facilitated transport in propagating therapeutic resistance within the cancer cell population. Utilizing endogenous exosomes for the targeted conveyance of treatment molecules, similar to miRNA or polypeptides, has developed as a potential tactic in cancer treatment. In a recent study, “cationic bovine serum albumin” (CBSA) was conjugated with “siS100A4”, specifically targeting the S100A4 protein associated with metastasis in TNBC. By using exosome-mediated RNA interference (RNAi), the expression of S100A4 protein was effectively down-regulated, resulting in significant inhibition of postsurgical metastasis in triple-negative breast cancer. These findings indicate the potential clinical efficacy of delivering “siS100A4” using core–shell structure enclosed within exosome membrane nanoparticles for cancer prevention and therapy. These findings indicate the potential clinical efficacy using core–shell structure enclosed within exosome membrane nanoparticles for cancer prevention and therapy [172, 173].

### Natural agent-based nano-carriers

These carriers play a significant role for creating anticancer medications. Phytochemicals derived from plants have shown the capacity to hinder carcinogenesis, the process of cancer formation as specified in Table 4. These plant-derived compounds possess unique chemical structures and bioactive properties that make them potential candidates for the creation of effective anti-cancer treatments. The exploration and utilization of phytochemicals offer promising avenues for the discovery of novel anti-cancer agents. Numerous bioactive natural substances have been recognized as potential inhibitors of metastasis by targeting various signalling pathways. These compounds hold significant promise in the field in the field of pharmaceutical exploration and advancement, as they contribute to the avoidance and management of metastatic cancer. Through modulation specific cellular signalling pathways, these bioactive natural compounds offer potential therapeutic benefits and lay the foundation for the creation of novel anti-metastatic drugs. Nanoparticles (NPs) have the ability to engage with various enzymes and proteins, leading to their biological activity on specific targets within the vast chemical space. Among the various bioactive compounds, phenolic compounds like flavonoids, lignin’s, tannins, and coumarins have been found to possess anticancer properties. These compounds exert their effects by triggering cell cycle arrest, inhibiting angiogenesis, inducing the tumour suppressor protein p53, and promoting apoptosis. A summary of these anticancer activities is provided in Table 4. The utilization of nanocarriers incorporating bioactive agents provides a wide array of potential uses in cancer treatment, enabling the targeted blocking of, specific routes. The physical and chemical characteristics of phytochemical components directly influence their drug absorption and distribution within the body. By encapsulating a drug within a nano transporter, a colloidal system is formed, which directly interacts with the biological system. One advantageous feature of nanocarriers is their capability to reduce the toxic effects commonly linked to the drug itself. An example of this is the combination of “tamoxifen” and “quercetin”, which, when enclosed in (“PLGA”) “polymeric nano transporters”, exhibited a reduction in breast tumour growth through the induction of apoptosis when administered orally. The application of quercetin-loaded nanoparticles made of polylactic acid (PLA) was investigated for the treatment of (BC) and exhibited notable reductions in tumour size and progression. Several additional studies have provided evidence that a diet rich in soy can have cancer-preventive effects due to the presence of the phenolic compound genistein (Gen). Genistein primarily exerts its effects by modulating apoptosis, angiogenesis, metastasis inhibition, and the cell cycle*.* An example of this is the combination of “tamoxifen” and “quercetin”, which, when enclosed in (“PLGA”) “polymeric nano transporters”, exhibited a reduction in breast tumour growth through the induction of apoptosis when administered orally. The application of quercetin-loaded nanoparticles made of polylactic acid (PLA) was investigated for the treatment of (BC) and exhibited notable reductions in tumour size and progression. Several additional studies have provided evidence that a diet rich in soy can have cancer-preventive effects due to the presence of the phenolic compound genistein (Gen). Genistein primarily exerts its effects by modulating apoptosis, angiogenesis, metastasis inhibition, and the cell cycle [174–176].

**Table 4:** Various phytoconstituents delivered through nanocarrier for cancer treatment

Phytoconstituent	Nanocarrier	Findings	Reference
Berberine concentrate	PLGA Nanoparticles	Cytotoxicity effects “MDA-MB 231”, “MCF 10A”, “MCF 7” of mammalian carcinoma	[177]
Curcumin	Nano lipid Carriers	Release pattern, photo cytotoxic anticancer effect on breast cancer cell	[178]
Curcumin	Solid Lipid Nanoparticles	Invitro Cytotoxic study on breast cancer cell lines	[179]
Curcumin & chrysin	Mesoporous Silica Nanoparticles	Cytotoxic effect on Olfactory Neuroblastoma cells	[180]
Resveratrol	Nanoparticles	Cytotoxic activity on (Caco-2)	[181]
Gallic acid	Fluorescein Isothiocynate Dyed MSN	Cytotoxic effect on Caco-2 cells	[182]

## Future prospects

The application of nanotherapeutics holds significant promise in effectively targeting and eradicating (BCSCs). These cells play a critical contribution to breast cancer development, recurrence, and resistance to conventional therapies like chemotherapy and radiation. By utilizing nanomedicine, there is a potential to address the challenges linked to BCSCs and improve the outcomes of breast cancer treatment. By utilizing nanomedicine, there is a potential to address the challenges linked to BCSCs and improve the outcomes of breast cancer treatment [183].

Combining cytotoxic agents in chemotherapy has demonstrated superior efficacy compared to using single agents. Nanomedicine, employing chemotherapeutic agents, offers a potent approach to specifically target breast cancer stem cells (BCSCs) by focusing on specific biomarkers such as CD44, D133, CD90, and ALDH**.** Additionally, nanomedicine effectively targets signalling pathway that has a critical function in sustaining BCSCs within cancer cells. This comprehensive strategy shows promise in addressing the challenges associated with BCSCs and provides a potential avenue for improved breast cancer treatment outcomes. The integration of conventional anticancer therapies with biomarkers has proven highly effective in specifically targeting and eliminating breast cancer stem cells (BCSCs). This combined approach exhibits superior efficacy against BCSCs compared to conventional therapies used alone. BCSCs are characterized by their elevated expression of CD44 receptors, transmembrane glycoproteins that serve as valuable surface markers for targeted therapeutic interventions. Combining cytotoxic agents in chemotherapy has demonstrated superior efficacy compared to using single agents. Nanomedicine, employing chemotherapeutic agents, offers a potent approach to specifically target breast cancer stem cells (BCSCs) by focusing on specific biomarkers such as CD44, D133, CD90, and ALDH. Additionally, nanomedicine effectively targets signalling pathway that has a critical function in sustaining BCSCs within cancer cells. This comprehensive strategy shows promise in addressing the challenges associated with BCSCs and provides a potential avenue for improved breast cancer treatment outcomes. The integration of conventional anticancer therapies with biomarkers has proven highly effective in specifically targeting and eliminating breast cancer stem cells (BCSCs). This combined approach exhibits superior efficacy against BCSCs compared to conventional therapies used alone. BCSCs are characterized by their elevated expression of CD44 receptors, transmembrane glycoproteins that serve as valuable surface markers for targeted therapeutic interventions [87, 184].

BCSCs exhibit CD44 surface markers and perform crucial functions in diverse cellular processes, such as “cell orientation”, “intercellular adhesion”, “cell–matrix signalling”, and “cell migration”. Researchers have targeted CD44 receptors using substances like “hyaluronic acid” (HA), “hyaluronan”, and “heparinase”. For example, they have utilized HA-functionalized nanoparticles (NPs) loaded with Salinomycin to specifically target BCSCs, inhibiting P-glycoproteins and potentially overcoming paclitaxel (PTX) resistance in cancer cells. Additionally, researchers have developed aptamer-conjugated nanoparticles (NPs) that show promising anticancer activity by effectively targeting and eliminating CD133 + BCSCs in both laboratory experiments and animal studies. Furthermore, ALDH activity, associated with drug resistance and increased tumorigenicity, is a crucial factor in BCSCs. Inhibition of “ALDH” activity through “NP-based drug delivery” has been investigated as an approach to surmount chemotherapy resistance in BCSCs. These approaches show the promise of nanoparticle-based drug delivery systems in targeting and combating BCSCs, providing valuable insights for breast cancer treatment [87].

In summary, researchers have investigated multiple approaches for targeting BCSCs, such as employing HA-functionalized nanoparticles to inhibit CD44 receptors, utilizing aptamer-conjugated nanoparticles for CD133 targeting, and employing NP-based drug delivery to inhibit ALDH activity. These strategies hold significant potential in overcoming chemoresistance and improving the effectiveness of breast tumour therapies. These strategies hold significant potential in overcoming chemoresistance and improving the effectiveness of breast tumour therapies [185].

As previously mentioned, targeting multiple signalling pathways involved in cancer proliferation, invasion, and migration holds significant potential in inhibiting tumour invasion and metastasis. Notably, inhibiting the “notch pathway” has been found to reduce the regeneration capacity of BCSCs, leading to reduced tumorigenic potential. Researchers have explored the use of γ-secretase inhibitors loaded onto mesoporous silica nanoparticles to effectively target and inhibit the notch pathway. This approach has shown promise in reducing the Breast CSC subpopulation and increasing the inclination of Breast CSCs to apoptosis induced by radiation therapy. These findings suggest a potential and promising strategy for combatting breast cancer and improving therapeutic outcomes. Likewise, scientists have investigated anthothecol-loaded PLGA nanoparticles (PLGA-NPs) as a means to down-regulate the Hedgehog signalling pathway, leading to a notable decrease in cell proliferation. This targeted approach highlights the potential of nanoparticle-based delivery systems in modulating specific signalling pathways. These discoveries present exciting prospects for the advancement of targeted cancer site using nanotechnology-based strategies. In a separate study, nanoparticles were employed to encapsulate siRNA and LY364947, a suppressor of the growth factor-beta signalling pathway. In a separate study, nanoparticles were employed to encapsulate siRNA and LY364947, a suppressor of an inhibitor of the growth factor-beta signalling pathway. The use of these nanoparticles resulted in the effective inhibition of the pathway, leading to tumour regression and a significant decrease in the proportion of BCSCs. This research highlights the promising potential of nanoparticle-based delivery systems in specifically targeting and suppressing the growth factor-beta signalling pathway, thereby yielding favourable outcomes in terms of tumour reduction and modulation of BCSCs. These findings provide valuable insights towards the development of nanoparticle-based treatment. These findings provide valuable insights towards the development of nanoparticle-based treatment [183, 185].

In the face of the challenges posed by (BC) and (TNBCs), a paradigm shift in treatment approach is necessary. Focusing on targeted elimination of tumour masses, specific subpopulations, and associated blood vessels through cellular targeting should take precedence. The future of BC treatment lies in personalized medicine, precisely tailored to each individual patient or unique cell subpopulation, for optimized therapeutic outcomes.

Advancing our knowledge of molecular biology and delving deeper into the complexities of Intercellular signalling in Drug resistance can open up exciting new avenues in BC treatment. Moreover, recognizing the critical function of the immune system in cancer defence, exploring the potential of plant-based antioxidants in cancer treatment, and understanding how traditional treatment methods bolster immunity are vital pursuits.

Immunotherapy has already shown great promise, but we must not overlook the significant contributions of hyperthermia-based approaches to the success of existing therapies. By comprehensively exploring these avenues, we can unlock their full potential in combating BC and TNBC.

Achieving functional and tailored therapy for each cancer patient demands a collaborative effort among molecular biologists, Phyto chemists, formulation scientists, and clinicians. This multidisciplinary alliance will pave the way for innovative treatment strategies that leverage the strengths of different fields, ultimately leading to improved patient outcomes and groundbreaking advancements in breast cancer treatment.

## Discussion

BC and TNBC present significant challenges in terms of targeted treatment and eradication. The key factors contributing to these challenges are varied immunogenicity, intra/inter-tumoral diversity, and the emergence of resistance due to epigenetic modifications or mutations. Although immunotherapy shows promise, its efficacy is currently limited to BC cases with higher immunogenicity, specifically HER2-positive and TNBC subtypes. The other BC subtypes typically display a limited immune profile and a reduced load of neoantigens. The presence of tumor heterogeneity within and between individuals, as well as at different tumor sites, presents a significant challenge in cancer therapy. Unique physical traits in different cell groups caused by variations in the cancer environment and the impact of epigenetic factors contribute to the malignancy of cancer. The ever-changing transitions among diverse cell subtypes within tumour’s create difficulties in determining the most suitable treatment for specific cellular groups.

The diverse tumor surroundings, combined with numerous signaling pathways within cancer cells, can also lead to the emergence of drug resistance. Phenotypic changes occurring in different Cell subsets may modify the responsiveness of specific cell types to treatments, making it challenging to effectively target and eliminate them. This intricate interplay between the tumor microenvironment, cellular heterogeneity, and signaling pathways further complicates the identification of optimal treatment strategies. While nanotherapeutics have shown promise in addressing the challenges of cancer therapy, some inorganic material-based nanomedicines have been associated with potential drawbacks. For instance, some nanomedicines have been found to induce the opening of endothelial channels, facilitating the transference of cancer cells into the vasculature. This unintended consequence can increase the risk of metastasis, a significant concern in cancer treatment. The process of lysosomal degradation can also compromise the stability and effectiveness of nanotherapeutic agents, limiting their therapeutic potential. Addressing these obstacles is crucial to optimize the delivery and efficacy of nanomedicine in cancer treatment.

BC and TNBC are particularly concerning due to their significant impact on female cancer patients' mortality rates. Their complexities, including the development of resistance, lack of primary receptors, and related side effects from existing therapies add complexity to effectively managing both the condition and its treatment. Combination therapies involving chemotherapy, surgery, and radiation are considered viable approaches to suppress tumors. However, despite this treatment regimen, relapse still occurs in a significant number of cases. Multifunctional micro-particles have surfaced as a hopeful framework for drug delivery in BC and TNBC. They offer diverse applications in cancer detection, imaging, and treatment. Microneedles provide an alternative approach for painless administration of anticancer agents, enhancing patient comfort during treatment. Both microspheres and microneedles hold significant potential in advancing the field of BC and TNBC management.

Nanotechnology-based systems represent a promising strategy for the effective transport of cytotoxic agents. Utilizing the distinct characteristics of nanoscale particles, including their Compact dimensions, substantial drug loading capacity, precise delivery capabilities, and robustness. when encapsulating active pharmaceutical ingredients, contributes to the enhanced effectiveness of drug delivery systems. Nanomedicines encompass a range of strategies, including The discovery of innovative compounds, including “nucleic acid-based therapeutics”, “monoclonal antibodies”, “phytoconstituents”, and “combined drug regimens”, provides a wide range of treatment options and progress in the nanomedicine domain. Gaining insights into the involvement of stem cells in tumor resistance, progression, and invasion offers multiple avenues to effectively manage tumor recurrence. Genetic material-based therapeutics, such as “siRNA”, “miRNA”, and “ncRNAs”, have been used to reduce cell proliferation, migration, invasion, and the spread of “BCSCs”. This targeted approach aims to promote recurrence- survival rate in cancer patients, providing a promising avenue for improved treatment outcomes. Additionally, clinical investigations are exploring the effectiveness of various chemotherapeutic drugs in combination with monoclonal antibodies, pathway inhibitors, and RNA-based therapies to surpass drug resistance and enhance treatment outcomes for breast cancer and TNBC patients.

Although various molecular targets have been identified for inhibiting There is scarce literature on the utilization of molecular targeted therapeutics in the context of nanomedicine. However, emerging evidence indicates that nanomedicine is a promising and innovative method in the field of cancer therapy, including breast cancer. Several studies in the literature highlight the potential of nanotechnology-based strategies for providing more selective and promising options in the identification and management of “breast cancer”. Ongoing research efforts continue to explore new avenues and innovative uses of nanotechnology, aiming to enhance the effectiveness and specificity of breast tumor therapy. The effective delivery of drugs and therapeutic agents to specific target sites is facilitated by the utilization of nanomedicine, which involves the conjugation of targeting selective moieties with nanoparticles (NPs). This approach enables precise and efficient drug delivery to the desired location. As a result, nanomedicine represents a highly promising strategy for the management of “breast cancer” “BC” in both the present and future. Its potential lies in its ability to improve effectiveness of treatments while minimizing adverse reactions, thus offering significant advancements in BC management and treatment outcomes.

## Conclusions

Nanomedicine has ushered in a transformative era in the diagnosis and treatment of both hormone-negative and hormone-positive breast cancer (BC). This revolutionary approach introduces a diverse range of nanoparticle-based formulations that have significantly impacted the management of BC. The future of breast cancer and triple-negative breast cancer therapies is poised to benefit from nanoparticles that seamlessly integrate therapeutic agents, molecular targeting, and imaging capabilities.

Their targeted approach aims to reach tumour stem cells through various mechanisms, preventing recurrence and transmission at the molecular level. This involves targeting specific receptors with nanocarriers formulated as inhibitors, providing a distinct advantage. The discussion encompasses various anti-stem cell therapies formulated as nanocarriers, demonstrating their efficacy in preventing recurrence. Emerging approaches, such as targeted formulation polymeric nanoparticles, metallic nanoparticles, liposomes, dendrimers, lipid-based drug delivery, and nucleic acid-based therapies, are explored to hinder unwanted cell proliferation. Additionally, nano-therapies designed specifically for tumour stem cell therapy are covered, showcasing the multifaceted applications of nanomedicine in cancer treatment.

Despite the promising potential of various therapeutic agents as potent inhibitors of TNBC, their application in nanomedicine has been relatively limited in existing research. However, with the deepening understanding of biological processes and continuous progress in nanotechnology, the future holds promise for the development of more compatible and efficient nanomedicine methods in the control of breast cancer and triple-negative breast cancer. This progress not only holds the potential to markedly enhance patient outcomes but also signifies a transformative shift in the approach to breast cancer management, paving the way for innovative developments in the field.

## Data Availability

Not applicable.
